# A Demonstration of Bromoform-Producing Gametophyte Culture for a Red Alga, *Asparagopsis taxiformis* in Laboratory Conditions

**DOI:** 10.1007/s10126-025-10493-2

**Published:** 2025-07-21

**Authors:** Ryuya Matsuda, Kazuyoshi Kuwano

**Affiliations:** 1 Fisheries Division, Japan International Research Center for Agricultural Science (JIRCAS), 1-1 Owashi, Tsukuba, Ibaraki 305-8686 Japan; 2 Faculty of Fisheries, Nagasaki University, 1-14 Bunkyo-Machi, Nagasaki, 852-8521 Japan

**Keywords:** Seaweed aquaculture, Seedling production, Life cycle, Tetrasporogenesis, Halogen metabolism

## Abstract

*Asparagopsis taxiformis* is a seaweed of interest for use as a livestock feed ingredient because of its high bromoform content, effectively reducing methane emissions from ruminants. A reliable protocol covering hatchery and nursery phases is essential to develop aquaculture techniques for gametophytes. The aim of this study was to establish a seedling production method for gametophytes under controlled laboratory conditions. We conducted the following: (1) induction of tetraspore release, (2) assessment of juvenile gametophyte growth, and (3) evaluation of gametophyte quality through bromoform quantification. Tetraspore release was successfully induced at 25 °C under an equinox photoperiod (12 h light: 12 h dark) or at 20 °C under short photoperiod (8 h light: 16 h dark), with initial spore release occurring after 12.3 ± 1.03 and 15.1 ± 1.26 days, respectively. Germinated tetraspores developed into 3–5 cm gametophytes over 2 months through static culture, followed by an aeration phase using natural seawater. Artificial seawater failed to promote gametophyte growth. Aeration improved the daily growth rate (6.86 ± 0.36%) compared to that of the static culture (4.72 ± 0.51%). Further cultivation promoted the development of main axes, lateral branches, and rhizomes. Bromoform was predominantly accumulated in the lateral branches (17.8 ± 12.3 mg·g^−1^ dry weight (DW)) and rhizomes (12.2 ± 1.37 mg·g^−1^ DW), compared to main axes (5.27 ± 1.95 mg·g^−1^ DW). Unlike tetrasporophytes, which constantly released bromoforms into the medium, gametophytes maintained stable extracellular bromoform levels. These findings demonstrate a feasible approach for laboratory-scale production of *A. taxiformis* gametophytes with consistent bromoform accumulation, contributing to the advancement of its aquaculture.

**Supplementary Information:**

The online version contains supplementary material available at 10.1007/s10126-025-10493-2.

## Introduction

Marine macroalgae produce a wide range of bioactive secondary metabolites with potential applications in agriculture, pharmaceuticals, cosmetics, and other industries owing to their antimicrobial, anti-inflammatory, and antifouling properties (Cabrita et al. 2010; Rocha et al. 2018; Carroll et al. 2024). Among red seaweeds (Rhodophyta), species in the genus *Asparagopsis* are notable for producing halogenated organic compounds, primarily bromoform, which have strong anti-methanogenic activity (Kladi et al. 2004; Paul et al. 2006a; Glasson et al. 2022). Recent studies have shown that feeding *Asparagopsis* spp. to ruminant livestock such as cattle and sheep significantly reduces methane emissions by inhibiting methanogenesis in the rumen microbiota (Machado et al. 2014, 2016; Li et al. 2016; Roque et al. 2019). As global efforts to reduce greenhouse gas emissions intensify, *Asparagopsis* has emerged as a promising feed additive and a potential driver of a new aquaculture-based methane mitigation industry.

Two species of *Asparagopsis*, *A. taxiformis* (Delile) Trevisan and *A. armata* Harvey, have attracted interest in cultivation. These species differ in thermal preferences: *A. taxiformis* is distributed from tropical to temperate waters, while *A. armata* prefers cooler seas (Ní Chualáin et al. 2004). Both often appear as invasive species (Zanolla et al. 2022a) and exhibit a triphasic heteromorphic life cycle consisting of carposporophytes, tetrasporophytes, and gametophytes (Zanolla et al. 2022a). Tetrasporophytes form small spherical clumps of filamentous branches (~ 2 cm), whereas gametophytes develop feathery fronds arising from stoloniferous branches (Bonin and Hawkes 1987; HasLin and Pellegrini 2001). *A. armata* gametophytes can be identified by the presence of barbed structures absent in *A. taxiformis* (Bonin and Hawkes 1987). These life stage-specific morphological traits influence their suitability for different cultivation systems; tetrasporophytes are more suited to land-based tank systems, whereas gametophytes are better adapted to sea-based farming (Schuenhoff et al. 2006; Mata et al. 2006).

Establishing effective aquaculture systems requires reliable protocols for seedling production. In *A. armata*, barbed gametophytes facilitate vegetative propagation (Wright et al. 2022), but *A. taxiformis*, which lacks this structure, requires tetraspore propagation by regulating tetrasporophyte sporogenesis. Environmental cues such as temperature and photoperiod are known to trigger sporogenesis in both species (Oza 1989; Guiry and Dawes 1992; Ní Chualáin et al. 2004; Mihaila et al. 2023; Theobald et al. 2024). Recent studies have demonstrated the successful induction of tetraspore release and germination of juvenile gametophytes in *A. armata* collected from New Zealand (Mihaila et al. 2024) and *A. taxiformis* collected from the Great Barrier Reef (Theobald et al. 2024). However, the development of nursery-stage cultivation methods remains underexplored. Some studies that attempt to cultivate wild gametophytes in the laboratory have failed (Ní Chualáin et al. 2004; Zanolla et al. 2022b), highlighting the need for research on optimal growth conditions and culture protocols. To date, most aquaculture-related studies on *Asparagopsis* spp. have been conducted in Oceania, particularly in Australia and New Zealand, whereas research in East Asia, including Japan, has been limited.

Another concern in commercial *Asparagopsis* cultivation is the accumulation of bromoform, the key compound responsible for methane inhibition (Machado et al. 2016). Bromoforms are stored in gland cells at both life stages (Paul et al. 2006a; Romanazzi et al. 2021). Although recent studies focused on bromoform accumulation in tetrasporophytes under controlled laboratory conditions (Hargrave et al. 2024; Resetarits et al. 2024; Torres et al. 2024), little is known about optimal conditions for bromoform accumulation in gametophytes. Understanding the patterns of bromoform accumulation is essential for evaluating product quality.

On the other hand, bromoform is considered one of very short-lived substances that possess catalytic potential to destroy the ozone layer in the troposphere and stratosphere (Zhang et al. 2020; Laube and Tegtmeier 2023). Although an estimate of the potential environmental impact of bromoform derived from *Asparagopsis* farming in Australia concluded that the effect on the ozone layer would be limited under normal operating conditions, the estimation was based on the data from tetrasporophytes of *A. armata* (Paul et al. 2006a; Jia et al. 2022). To our knowledge, no studies have compared bromoform emission activity between gametophytes and tetrasporophytes. Elucidating the mechanism of bromoform leakage from *Asparagopsis* spp. is essential for the improvement of cultivation techniques.

The aim of this study was to establish a method for culturing *A. taxiformis* gametophytes originating in Japan and to provide fundamental physiological insights revalent to *Asparagopsis* farming. We (1) evaluated the effects of temperature and photoperiod on tetraspore induction, (2) developed a nursery protocol for gametophyte seedling growth, and (3) assessed the intercellular accumulation and extracellular emission of bromoform in laboratory-cultured gametophytes.

## Materials and Methods

### Algal Material and Culture Conditions

Tetrasporophytes of *Asparagopsis taxiformis* used in this study were derived from gametophytes originally collected from the beach of Minamishimabara, Nagasaki, Japan (32°36′37″N, 130°13′37″E) during low tide on 4 July 2023. Mature gametophytes containing carposporangia were collected and stored in autoclaved seawater under dim-light conditions. Carpospores released 3 days after collection were isolated using a glass pipette with a fine tip under an inverted microscope (CKX41, Olympus Co., Ltd., Japan) and inoculated into each well of a 24-well plate. Early tetrasporophytic thalli, without other algae, fungi, or protozoans, were carefully selected and transferred to glass flasks. The tetrasporophytic thalli were maintained as stock cultures in glass flasks or jars (500 mL to 8 L) filled with autoclaved natural seawater enriched with a nutrient mixture (described below). Cultures were kept at a constant temperature of 20 °C under a 12:12 light:dark photoperiod (10–15 µmol m⁻^2^ s⁻^1^), with filtered aeration (Minisart SRP syringe filter, 0.45 µm, Sartorius AG, Germany). The base medium was sand-filtered natural seawater (34–35‰ salinity) obtained from the Ibaraki Prefectural Government Fishery Research Institute and sterilized by autoclaving at 121 °C for 20 min.

The nutrient mixture consisted of 100 mM sodium nitrate, 5 mM sodium phosphate, and 1 mM iron sulfate, chelated with 1 mM EDTA. This stock solution was diluted 1:1000 in the culture medium, resulting in final concentrations of 100 µM nitrate, 5 µM phosphate, and 1 µM iron/EDTA. This composition was chosen because it enabled contamination-free sporophyte cultures. The nutrient levels were approximately 1/8 strength Provassoli's enriched seawater (PES) medium (824 µM nitrate, 46.3 µM phosphate; Provasoli 1968). All media, glassware, and other heat-resistant equipment were sterilized by autoclaving at 121 °C for 20 min.

**Fig. 1:**
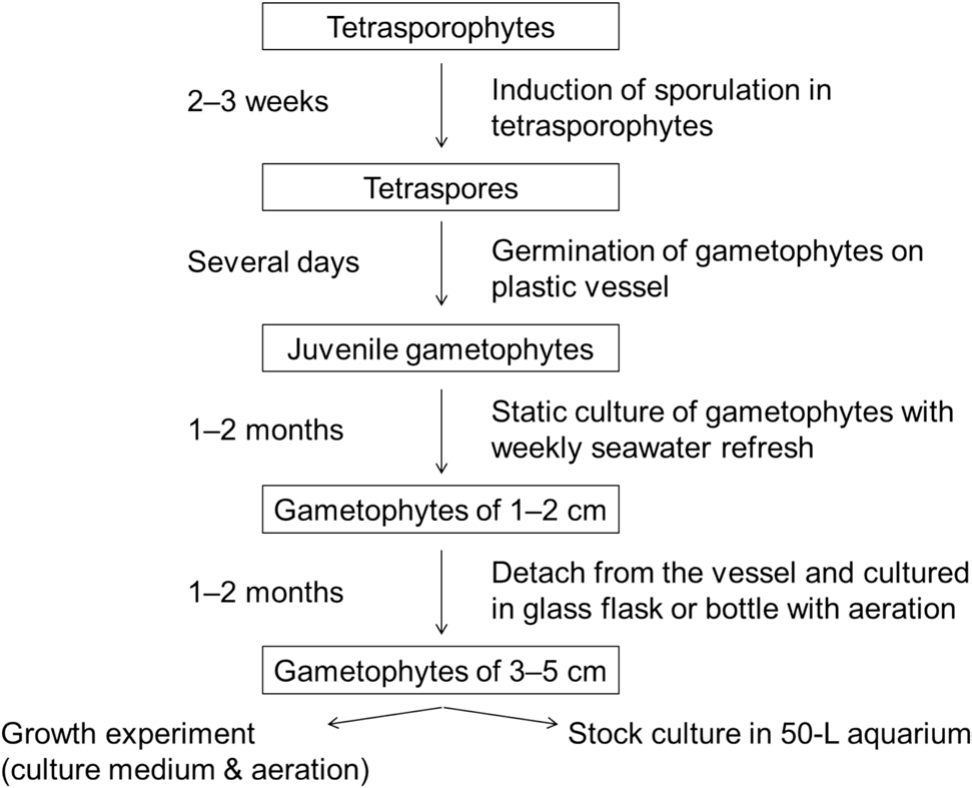
Schematic of gametophyte production and experimental design in this study. Stocked tetrasporophytes were used in sporulation experiments (Fig. 2). The released tetraspores then germinated and developed into gametophytes. Gametophytes cultured in flasks were subsequently used in culture experiments (Figs. 3 and 4) and maintained as stock cultures for bromoform experiments (Figs. 5 and 6)

Tetrasporophytes in stock cultures formed spherical clumps and were maintained at a biomass density of approximately 1–2 g/L (Fig. S1a). For tetraspore production, tetrasporophytes were transferred to fresh vessels (see below). Released tetraspores were settled in 150 mL plastic cups, and the germinated juvenile gametophytes were maintained at 20 °C under the same light conditions (10–15 µmol m⁻^2^ s⁻^1^, 12:12 L:D) without aeration for 1 month. The medium was refreshed weekly using the same vessel to retain attached juveniles. When gametophytes reached 0.5–1 cm in length, they were detached using sterilized tweezers and transferred to 500 mL or 1 L glass flasks with aeration for 1 month. The aerated gametophytes grew to approximately 4 cm without an apparent morphological distinction between shoots and rhizomes. A portion of these individuals was used in a comparative growth experiment, while the remaining individuals were transferred to 50 L acrylic tanks filled with enriched autoclaved seawater under gentle aeration. During this phase, gametophytes became entangled, but well-developed fronds (Fig. S1b). Some individuals had developed cystocarps and spermatangia (Fig. S1c–d). Immature gametophytes were selected for further experiments. A schematic of gametophyte production and experiments is shown in Fig. 1.

### Sporulation Experiment

Formation of tetrasporangia (Fig. S1e–f) and the release of tetraspores (Fig. S1g) were induced by culturing tetrasporophytes at different temperatures (20, 25, and 30 °C) or photoperiods (14:10, 12:12, 10:14, and 8:16 light:dark), each tested independently. Temperature experiments were conducted in a temperature-controlled incubator (TG300WLED-3LE, Nippon Ikakikai Seisakusho Co., Ltd., Japan) with a built-in white LED light source operating under a 12:12 L:D cycle. The photoperiod experiment was conducted using white plastic buckets (L 33.5 × W 21 × H 31.5 cm) illuminated by an overhead LED light (LED Power II 300, GEX Co., Ltd., Japan), maintained at 20 °C.

Tetrasporophytes were weighed (10–30 mg fresh weight), placed in 30 mL of enriched sterilized seawater, and cultured in 1-oz A-PET plastic cups (Heiko Pack Co., Ltd., Japan). Twelve replicate cups were prepared for each treatment group. Light intensity was set to 13 µmol m⁻^2^ s⁻^1^ using a portable photon meter (MQ-200X, Apogee Instruments, USA).

During the 21-day culture period, the seawater medium was refreshed every 3 days. The fresh weight of the algal tissues was recorded at each change. The algal mass was lifted with sterilized tweezers from medium and gently blotted with Kimwipes (Nippon Paper Crecia Co., Ltd., Japan) repeatedly to remove excess moisture. The number of released tetraspores that settled at the bottom of each cup was counted daily under a microscope. The plastic cup was replaced each day to facilitate daily spore observation; however, the same seawater medium was used for 3 days.

The mean initial day for the detection of tetraspores in each replicate unit (plastic cup) was calculated (*n* = 12) to evaluate the timing of sporulation. In addition, the culture duration required for 50% of the replicate units to release tetraspores was estimated as 50% probability of spore release. Spore count data were normalized to the fresh weight of the algal tissue in each unit (see Data analysis section).

### Culture Experiment for Gametophyte Growth

Thalli previously cultured in glass flasks were transferred to 300 mL plastic cups to evaluate the effect of different seawater qualities on gametophyte growth. Three seawater treatments were prepared: natural seawater (N/SW), which was also used in the standard culture protocol; artificial seawater (A/SW), which was prepared by dissolving Marine Art SF-1 (Osaka Yakken Co., Ltd., Japan) in purified water (filtered using a G-10D cartridge water purifier, ORGANO Corp., Japan); and a 1:1 mixture of N/SW and A/SW (N + A/SW). All seawater media were autoclaved and enriched with the same nutrient mixtures used for the stock cultures. The culture vessels were maintained under the same conditions as the stock cultures (20 °C, 12:12 light:dark cycle, 10–15 µmol m⁻^2^ s⁻^1^, with filtered aeration). Fresh weight was recorded at each medium change, every 3 days for the first 15 days, and every 6 days from day 15 to day 33.

Thalli previously cultured in glass flasks were transferred to 500 mL flasks to evaluate the effect of aeration on gametophyte growth. Two treatments were established: with and without aeration. Fresh weight and seawater medium were recorded and exchanged every 7 days. Gametophytes were photographed on a Petri dish using a digital camera (Tough TG-6, Olympus Corporation, Japan) after 28 days of culture, and then stored at − 80 °C until bromoform extraction. The digital images were edited for white balance using GIMP version 2.10.36 (https://www.gimp.org/), and cropped as needed.

Growth was expressed as the daily growth rate (DGR), following the method described by Yong et al. (2013):

$$\text{DGR} (\%)=\left[{\left(\frac{{FW}_{t}}{{FW}_{0}}\right)}^\frac{1}{t}-1\right]\times 100$$

where *FW*_*t*_ is the fresh weight (mg) at time *t* (day) and *FW*_*0*_ is the initial fresh weight (mg).

### Division of Gametophyte Thallus to Parts

Some individuals in the gametophyte stock culture developed clearly distinguishable fronds (shoots) and rhizome structures. Immature individuals were gently untangled and dissected into shoots and rhizomes using sterile tweezers.

The boundary between the shoot and rhizome was defined as the region between the lowest point of the major branches and the first branch of the rhizome. Fronds were further divided into erect main axes and lateral branches. The rhizome, composed of stolon-like stems and spiny branches, was not further subdivided owing to the complexity of separation. Each dissected part was weighed separately and stored at − 80 °C for later analysis.

### Sample Preparation for Bromoform Analysis

Algal samples were stored at − 80 °C until freeze-drying. Freeze-drying was performed in a 30 L desiccator (AS ONE Corp., Japan) connected to a freeze dryer (FDU-2100, Tokyo Rikakikai Co., Ltd.) for 2 days. All samples were freeze-dried simultaneously. After freeze-drying, the dry weight of each sample was measured and samples were ground in a mortar with 5–10 mL of methanol. The supernatant was collected by centrifugation at 20,000 × *g* for 5 min at 4 °C. Methanol extracts were transferred into 2 ml autosampler vials, sealed without air inclusion and stored at 4 °C until analysis. All methanol extractions were performed on the same day.

Gametophytes and tetrasporophytes from stock cultures were incubated in 100 mL of seawater under standard culture conditions without aeration, to prepare seawater samples containing the released bromoforms. Three replicates were prepared for each treatment group. Seawater samples were collected every 2 days over a period of 8 days (i.e., on days 0, 2, 4, 6, and 8). Each culture cup was gently swirled once daily to homogenize bromoform concentrations. Seawater samples were transferred into 40 ml purge and trap vials, sealed without air inclusion and stored at 4 °C until analysis.

### Bromoform and Water Content

Methanol extracts and seawater samples were analyzed using outsourced gas chromatography–mass spectrometry (GC–MS), following Matsuda et al. (2015), with minor modifications. For methanol extracts, a PerkinElmer Clarus SQ8T GC–MS system equipped with an Elite 624 column (PerkinElmer, USA) was used. The temperature program was as follows: initial temperature of 70 °C (1 min), ramped at 20 °C/min to 200 °C, and held for 4 min. Helium was used as the carrier gas. The limit of detection (LOD) and limit of quantification (LOQ) were 0.03 mg/mL and 0.1 mg/L, respectively. The recovery rate was 95–98%.

The moisture content (MC) and bromoform yield (BY) were calculated using the following equations:

$$MC (\%) =\left(1- \frac{DW}{FW}\right) \times 100$$

$$BY (\mu g/mg) = \frac{BF_{MeOH} \times {V}_{MeOH}}{DW}$$

where DW and FW are the dry and fresh weights (mg) before and after freeze-drying, respectively, *BF*_*MeOH*_ is the bromoform concentration in the methanol extract (µg/mL), and *V*_*MeOH*_ is the volume used for extraction (mL).

Seawater samples were analyzed using a PerkinElmer TurboMatrix 40 Trap system with a GL Science AQUATIC column (PerkinElmer, USA). The temperature program started at 40 °C (1.5 min), followed by a ramp of 10 °C/min to 200 °C (4 min). The LOD and LOQ were 0.06 mg/mL and 0.2 mg/L, respectively, and the recovery rate was 96–99%.

Bromoform yield (BY) from seawater were calculated as:

$$BY (\mu g/mg) = \frac{BF_{SW} \times {V}_{SW}}{DW}$$

where DW is the dry weight (mg) of seaweed after freeze-drying, *BF*_*SW*_ is bromoform concentration in the seawater (µg/mL), and *V*_*SW*_ is the volume of seawater remaining (100 mL).

### Data Analysis

Statistical analyses were conducted primarily using generalized linear models (GLMs) and generalized linear mixed-effects models (GLMMs). To estimate the probable culture period at which 50% of tetraspore release, binomial logistic regression was performed using the glmer function in the R package “lme4” (Bates et al. 2015). The culture vessel was treated as the unit of replication, with the culture period (in days) as the explanatory variable and spore release probability as the response. If one or more tetraspores were observed on a given day, the spore release probability was recorded as 1; if no tetraspores were observed, it was recorded as 0. Replicates were incorporated as random intercepts.

Tetraspore counts were normalized by dividing the number of spores released by the estimated fresh weight of the tetrasporophytes in each vessel. Fresh weight was estimated using linear regression, where the explanatory variable was the culture day and the response was algal weight. Individual growth curves and regression models are shown in Supplementary Figures (Fig. S2).

Gamma regression models (glm in R) were used to analyze gametophyte growth and bromoform content. Each culture vessel was treated as a unit of replication (*n* = 4). For the aeration experiment, aeration conditions were used as the explanatory variable, daily fresh weight as the response variable, and initial weight (FW_o_) as the offset (Pinheiro and Bates 2000; Saito et al. 2023). The moisture content data were analyzed using a binomial regression model (glm in R). Bromoform content was also analyzed using gamma regression, with bromoform amount as the response variable and dry weight (DW) as an offset. For multiple comparisons, Tukey’s honestly significant difference (HSD) test was performed using the comp function in the R package “multicomp” (Hothorn et al. 2008).

A significance level of *p* ≤ 0.05 was used to determine statistically significant differences. All statistical analyses were conducted using R software version 4.4.1 (R Core Team 2024).

## Results

### Sporulation from Tetrasporophytes

Tetraspores were released from tetrasporophytes cultured at 25 °C under a 12 h light:12 h dark (12:12 L:D) photoperiod and at 20 °C under a short-day condition of 8 h light:16 h dark (8:16 L:D). No mature cells (i.e., tetrasporangia) were observed under other conditions, including 20 °C and 30 °C with 12:12 L:D (Table 1), or under longer photoperiods (10:14, 12:12, and 14:10 L:D) at 20 °C (Table 2).

**Table 1:** Effect of temperature on tetraspore release

Temperature	Photoperiod	Tetraspore release
20 °C	12:12 L:D	-
25 °C	12:12 L:D	+
30 °C	12:12 L:D	-

**Table 2:** Effect of photoperiod on tetraspore release

Temperature	Photoperiod	Tetraspore release
20 °C	8:16 L:D	+
20 °C	10:14 L:D	-
20 °C	12:12 L:D	-
20 °C	14:10 L:D	-

**Fig. 2:**
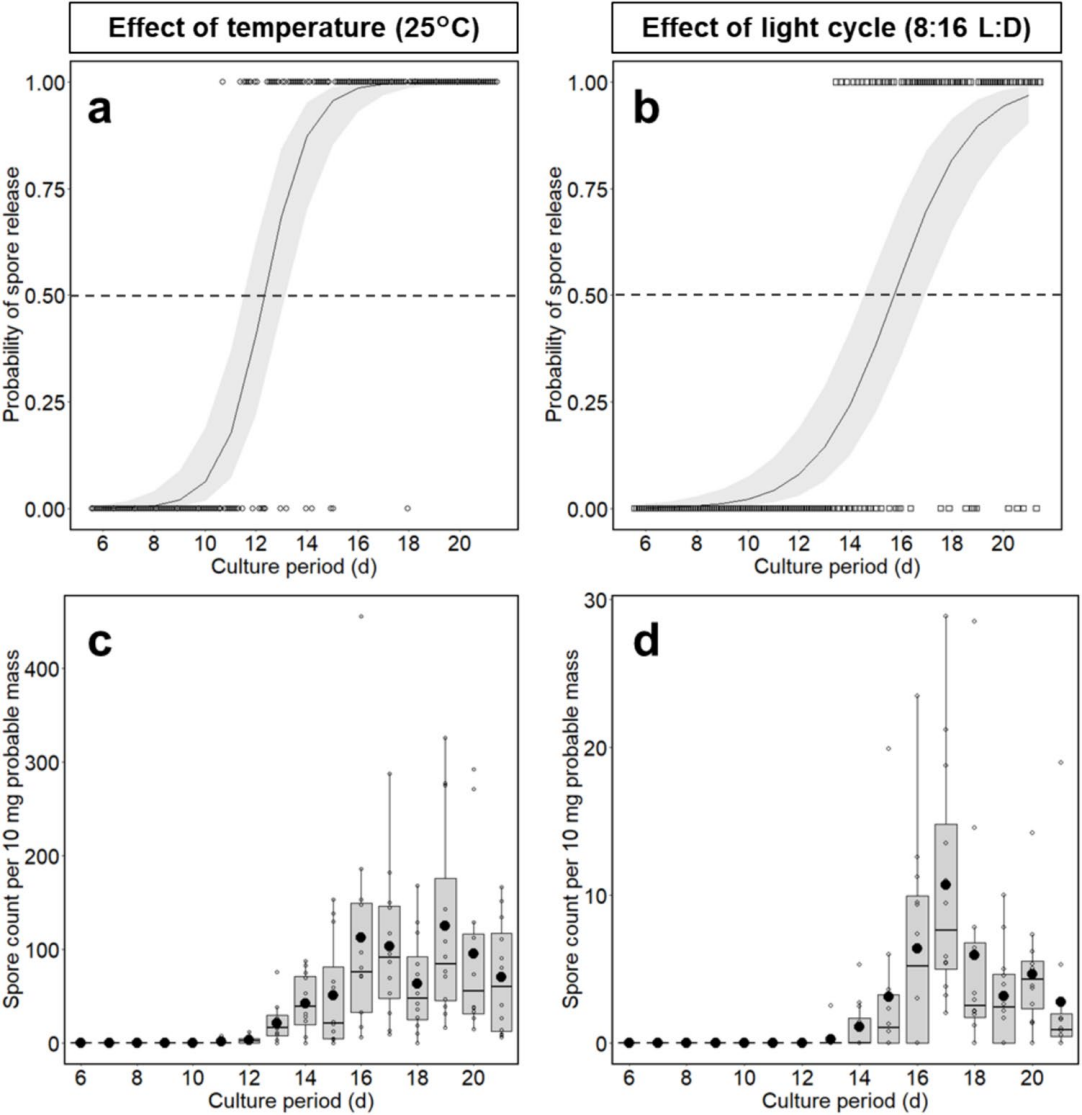
Sporulation of tetrasporophytes. Tetrasporophytes were incubated for 21 days at an elevated temperature (25 °C) or short photoperiod (8:16 L:D) (*n* = 12). The probability of tetraspore release was modeled for temperature treatment (**a**) and photoperiod treatment (**b**). White circles (a) and squares (b) represent the daily spore release probability, scored as 1 when one or more spores were released, and 0 when none were observed. Curves show the predicted probabilities with 95% confidence intervals (shaded areas) generated using the GLMM. Dotted lines indicate 50% probability of spore release. Boxplots display the number of spores released daily under temperature (**c**) and photoperiod (**d**) conditions. White circles (c) and squares (d) represent individual replicate measurements. The black dots represent the daily mean number of spores released

The average time required for initial spore release was 12.3 ± 1.03 days at 25 °C and 15.1 ± 1.26 days under the short-day condition. These values closely matched the estimated 50% probability of spore release at 12.3 and 15.7 days, respectively (Fig. 2a, b).

At 25 °C, the mean daily spore release was 6.69 ± 5.12 and 62.0 ± 48.9 spores/mg of tetrasporophyte during the second (days 8–14) and third (days 15–21) weeks, respectively (Fig. 2c). In contrast, under the short-day condition (20 °C, 8:16 L:D), the respective values were much lower, at 0.128 ± 0.198 and 3.66 ± 2.65 spores/mg (Fig. 2d). We also confirmed that a combined treatment of 25 °C and 8:16 L:D successfully induced sporulation (Fig. S3). In this experiment, we compared the required time for the first tetraspore release in a replicate unit between short (8:16 L:D) and equinox (12:12 L:D) photoperiod. The short photoperiod appeared to facilitate earlier spore release (11 ± 0.73 days) than the equinox period (12.9 ± 0.79 days). However, the difference was not statistically significant (*P* > 0.05), based on a Poisson model. In contrast, tetraspore release was not observed in artificial seawater (A/SW) under conditions of elevated temperature (25 °C) and short photoperiod (8:16 L:D) for 21 days (data not shown).

The total number of released spores over the 21-day culture period was 1,203 ± 844.5/mg under the temperature treatment (25 °C) and 67.2 ± 37.3/mg under the short photoperiod condition. Although the germination rate of tetraspores was not quantified in this study, a sufficient number of juvenile gametophytes were successfully obtained and cultured for subsequent experiments.

### Effect of Different Culture Media on Gametophyte Growth

**Fig. 3:**
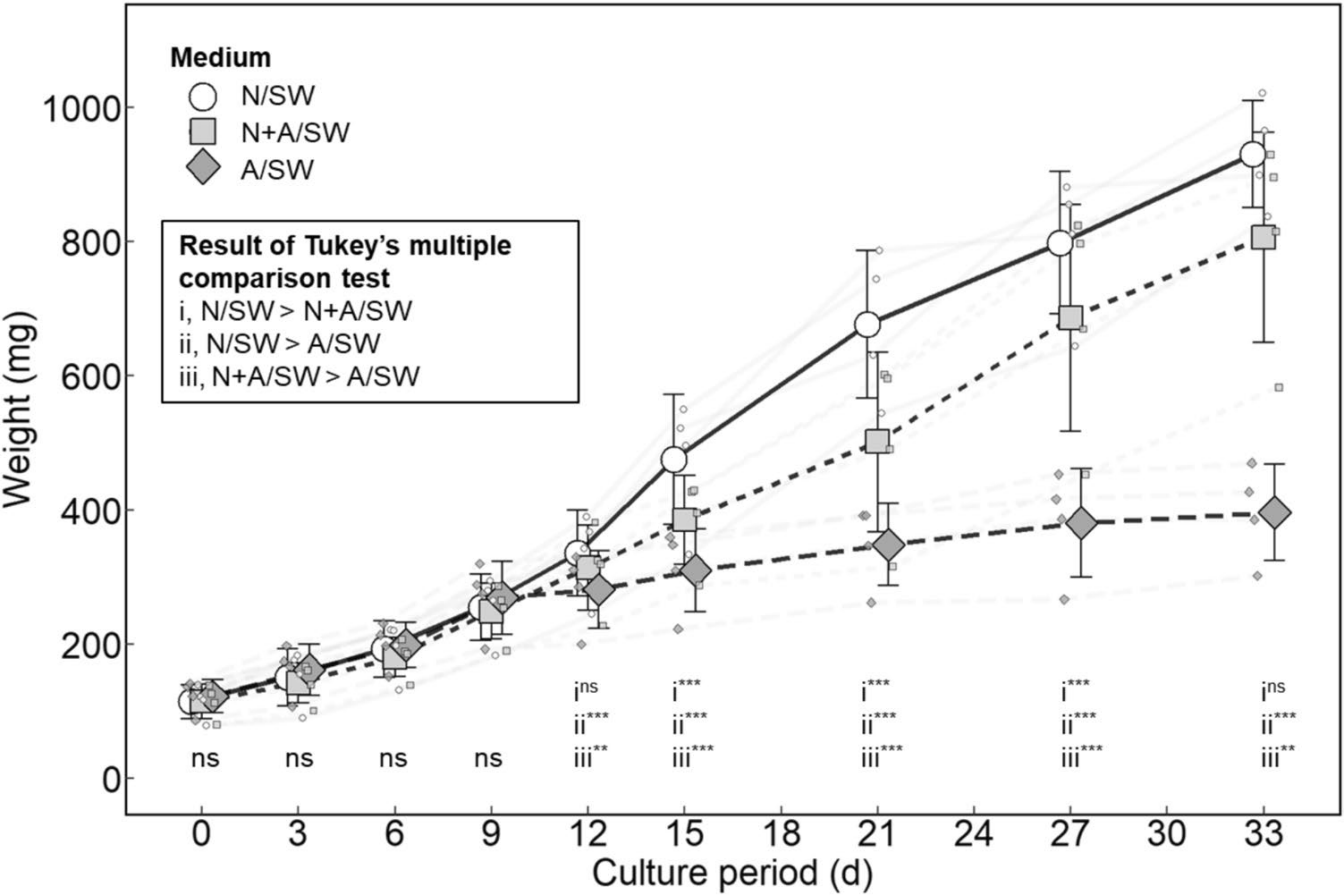
Effect of culture media on gametophyte growth. The gametophytes were cultured in natural seawater (N/SW), artificial seawater (A/SW), or mixed seawater (N + A/SW). Growth curves show mean values and standard deviations of fresh weight at each time point. Small circles, squares and diamonds indicate individual replicate values. (*n* = 4). Statistical analysis was performed using GLM for the three seawater types at each time point, followed by Tukey's HSD test for multiple comparisons when significant differences were detected. Group differences are indicated as i, ii, and iii (ns: not significant; *P < 0.05; **P < 0.01; ***P < 0.001)

Gametophytes cultured in different seawater types exhibited similar growth across all treatments during the first 9 days (*P* > 0.05), but significant differences emerged thereafter (*P* < 0.001; Fig. 3). The highest growth was observed in natural seawater (N/SW), whereas mixed seawater (N + A/SW) supported comparable growth. In contrast, artificial seawater led to reduced growth after day 9. Statistical analysis revealed that from day 12 onward, the biomass of gametophytes cultured in A/SW was significantly lower than that of those cultured in N/SW and N + A/SW (*P* < 0.01). During the initial 9-day period, the average daily growth rate (DGR) was 9.44 ± 0.87% in N/SW, 9.15 ± 0.69% in N + A/SW, and 9.23 ± 0.32% in A/SW (Fig. S4). The average DGR over the full 33-day period was 6.63 ± 0.57% in N/SW, 6.11 ± 0.11% in N + A/SW, and 3.66 ± 0.14% in A/SW (Fig. S4).

### Effect of Aeration on Gametophyte Growth and Bromoform Content

**Fig. 4:**
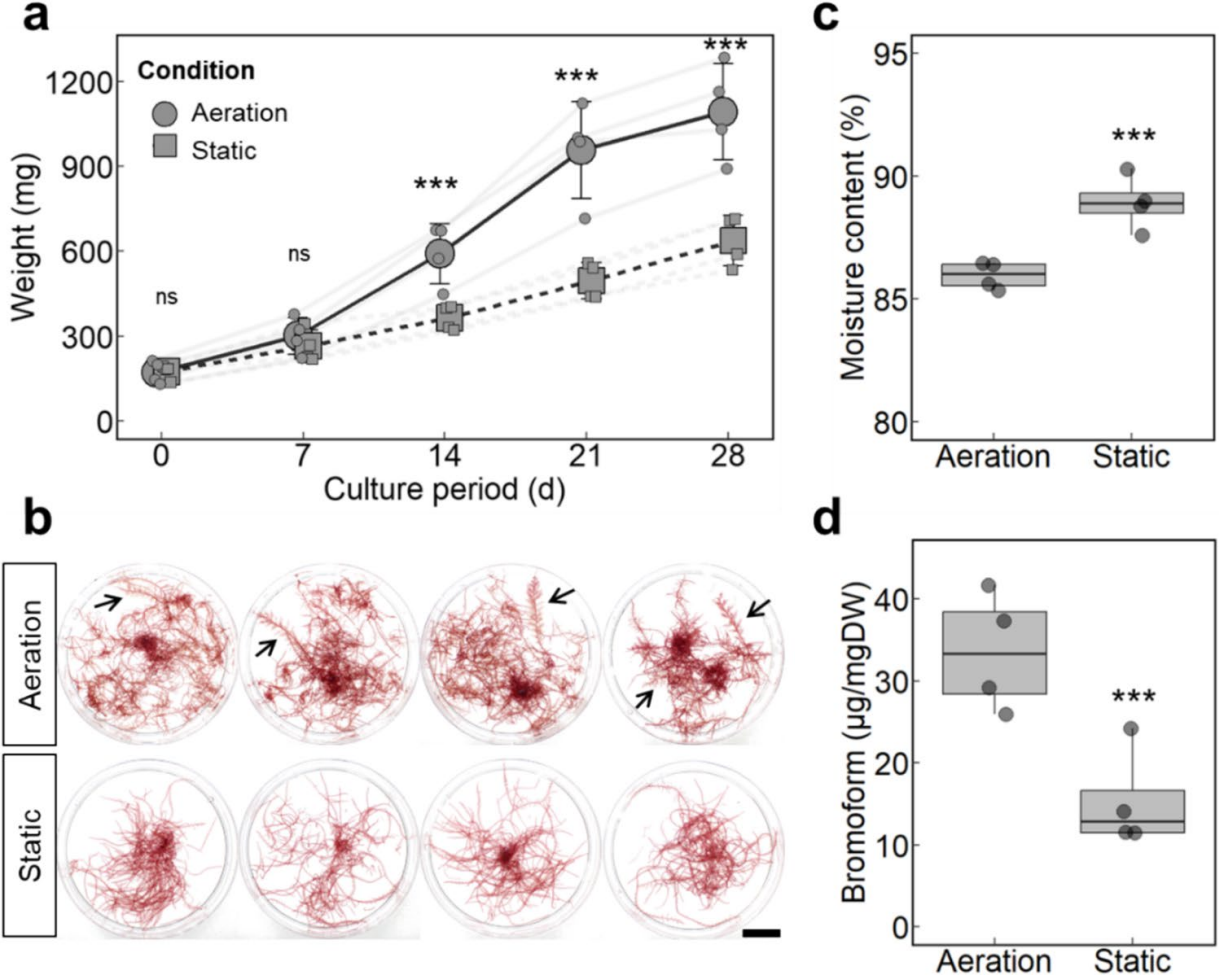
Effect of aeration on gametophyte growth and bromoform accumulation. Gametophytes were cultured for 28 days under aerated and non-aerated (static) conditions (*n* = 4). (**a**) Growth curves showing mean fresh weight and standard deviation. Small circles and squares indicate individual replicate values. (**b**) Photographs of gametophytes after 28 days of culture; arrows indicate developed shoot structures; scale bar indicates 2 cm. (**c**) Moisture content after 28 days is shown in a boxplot. (**d**) Bromoform content on a dry weight basis is shown in a boxplot. Statistical analysis was performed using a GLM (ns: not significant; ***P < 0.001)

Algal biomass was significantly higher in aerated cultures than in static cultures after 14 days (*P* < 0.001; Fig. 4a). The DGR averaged 6.86 ± 0.36% with aeration and 4.72 ± 0.51% under static conditions over the 28-day culture period. Aeration promoted the development of distinct shoot morphologies, including shoot axes and densely entangled rhizomes, whereas static culture resulted in elongated axes with small branches (Fig. 4b).

Moisture content was significantly lower in aerated cultures (86.0 ± 0.56%) than in static cultures (88.9 ± 1.11%) (*P* < 0.001; Fig. 4c). In addition, bromoform content on a dry-weight basis was significantly higher in aerated treatments (33.5 ± 7.26 mg·g⁻^1^ DW) than in static treatment (15.3 ± 6.03 mg·g⁻^1^ DW) (*P* < 0.001; Fig. 4d).

### Bromoform Accumulation in Gametophytes

**Fig. 5:**
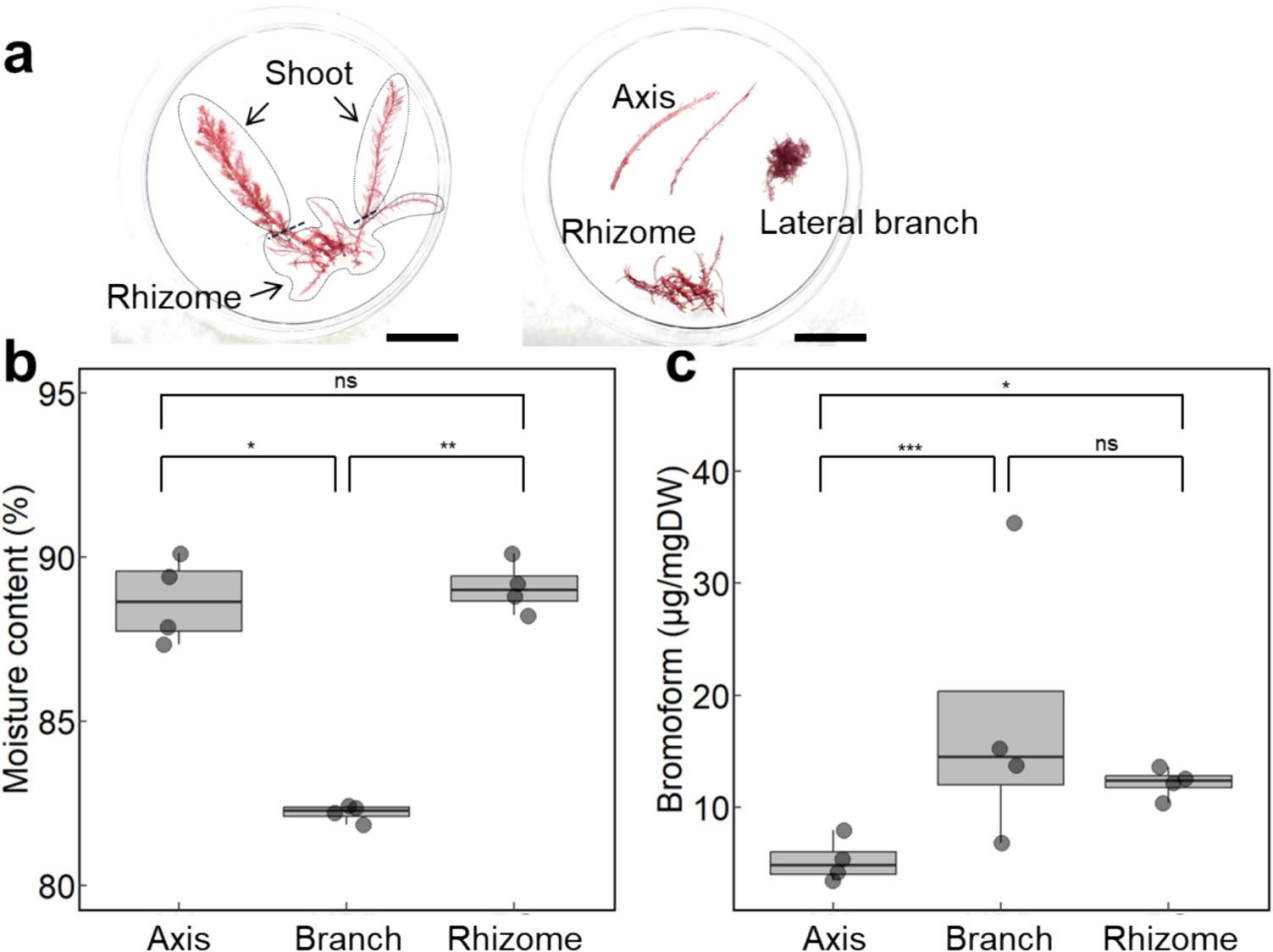
Distribution of bromoform in gametophyte tissues. Stock-cultured gametophytes without visible reproductive structures were selected and dissected into shoot, lateral, and remaining rhizomes (*n* = 4). (**a**) Representative gametophytes photographed before and after dissection; scale bars = 2 cm. (**b**) The moisture content of the three tissue types is shown in a boxplot. (**c**) Box plot showing bromoform content on a dry weight basis for each tissue type. Statistical analysis was performed using a GLM, followed by Tukey's HSD test for multiple comparisons (ns: not significant; *P < 0.05; **P < 0.01; ***P < 0.001)

Well-developed gametophytes were dissected into the main axes, lateral branches, and rhizomes to investigate the tissue-specific bromoform distribution (Fig. 5a). Lateral branches exhibited significantly lower moisture content (82.2 ± 0.26%) than both main axes (88.7 ± 1.29%, *P* = 0.014) and rhizomes (89.1 ± 0.79%, *P* = 0.002), while no significant difference was observed between the latter two (*P* > 0.05; Fig. 5b).

Bromoform was detected in all parts, but dry-weight-based concentrations were significantly higher in lateral major branches (17.8 ± 12.3 mg·g⁻^1^ DW) and rhizomes (12.2 ± 1.37 mg·g⁻^1^ DW, *P* < 0.001) than in main axes (5.27 ± 1.95 mg·g⁻^1^ DW, *P* = 0.025). No significant differences were observed between branches and rhizomes (*P* = 0.466; Fig. 5c). On a fresh-weight basis, the lateral branches showed a significantly higher bromoform content than both the main axes (*P* < 0.001) and rhizomes (*P* = 0.013) (Fig. S5).

### Bromoform Emission in Gametophytes and Sporophytes

**Fig. 6:**
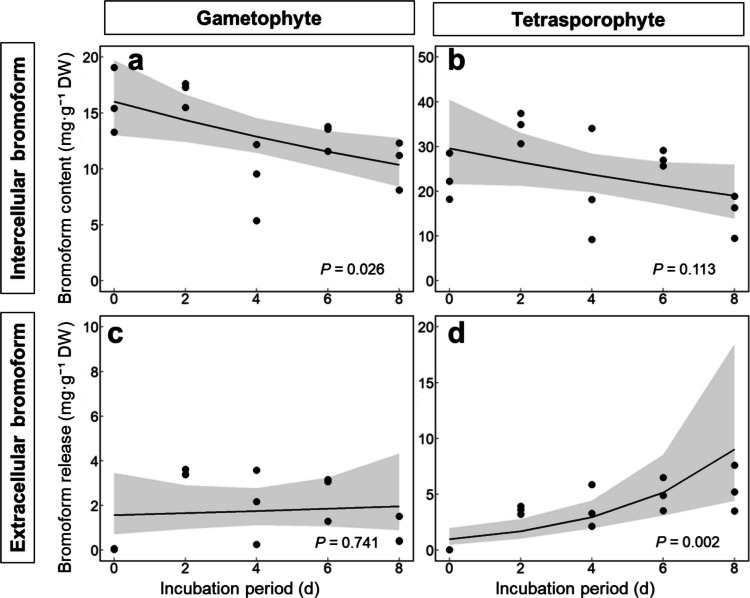
Intercellular and extracellular bromoform concentrations in gametophytes and tetrasporophytes. Gametophytes and tetrasporophytes were incubated in seawater for 0, 2, 4, 6, and 8 days (*n* = 4). Intercellular bromoform contents of gametophytes (**a**) and tetrasporophytes (**b**). Bromoform concentrations in the surrounding seawater for gametophytes (**c**) and tetrasporophytes (**d**). Regression curves and their 95% confidence intervals (shaded areas) were generated using a GLM, with incubation period as the explanatory variable

Bromoform production and emission patterns were compared between gametophytes and tetrasporophytes (Fig. 6). The intracellular bromoform content showed a significant trend over the incubation period in gametophytes (*P* = 0.026; Fig. 6a) but not significant in tetrasporophytes (*P* = 0.113; Fig. 6b). Similarly, the bromoform concentration in seawater incubated with gametophytes remained stable throughout the experiment (*P* = 0.741; Fig. 6c), suggesting that gametophytes suppressed bromoform release under normal conditions and prevent continuous emission into the surrounding medium. In contrast, seawater from the tetrasporophyte incubations exhibited a significant increase in bromoform concentration during this period (*P* = 0.002; Fig. 6d), indicating that the tetrasporophytes continuously released bromoform during the incubation period. A simple calculation of the hourly bromoform release rate between days 2 and 8 revealed wide range in gametophytes (1.860–74.468 µg g^−1^ DW h^−1^) and tetrasporophytes (18.168–81.331 µg g^−1^ DW h^−1^). The mean values, without accounting for temporal trends, were 28.128 ± 26.815 µg g^−1^ DW h^−1^ for gametophytes and 43.920 ± 20.743 µg g^−1^ DW h^−1^ for tetrasporophytes.

## Discussion

The production of gametophyte seedlings is a critical step in developing aquaculture techniques for *Asparagopsis taxiformis*. Previous studies have successfully induced tetraspore reproduction (hatchery phase) using *Asparagopsis* spp. collected from the Southern Hemisphere (Mihaila et al. 2023; Theobald et al. 2024). Furthermore, those studies focused on the growth of juvenile gametophytes immediately after germination (early nursery phase) (Mihaila et al. 2024; Theobald et al. 2025). In the present study, we developed seedling production techniques for the hatchery phase using a strain of *A. taxiformis* collected in Japan and evaluated the growth and bromoform content in well-developed gametophytes during the nursery phase. We demonstrated that (1) a temperature (25 °C) and a short photoperiod (8:16 L:D) induced sporulation well (Fig. 2); (2) juvenile gametophytes required component present in natural seawater and benefited from aeration for morphological development (Figs. 3 and 4); and (3) bromoform accumulated more in lateral branches than in main axes, with a certain level of extracellular emission (Figs. 4, 5 and 6). These findings are discussed in detail below.

### Sporulation and Germination

The effects of temperature and photoperiod on sporulation in *Asparagopsis taxiformis* and *A. armata* have been examined in previous studies (Oza 1989; Guiry and Dawes 1992; Ní Chualáin et al. 2004; Mihaila et al. 2023; Theobald et al. 2024; summarized in Table S1). In those studies, the effect of temperature was primarily examined under a fixed short photoperiod (8:16 L:D). In the present study, we tested the effects of temperature and photoperiod independently while maintaining other factors under standard stock culture conditions. Sporulation was successfully induced at 20 °C under a short photoperiod (8:16 L:D) and at 25 °C under an equinox photoperiod (12:12 L:D) (Tables 1 and 2). Our results under the short-day condition (20 °C, 8:16 L:D) were consistent with previously reported sporulation temperatures under the short photoperiod for *A. taxiformis* from Shimane, Japan (17–27 °C); Hawaii, USA (17–29 °C) (Ní Chualáin et al. 2004); and Queensland, Australia (19–25 °C) (Theobald et al. 2024). In contrast, sporulation under an equinox photoperiod (12:12 L:D) has not been previously assayed in *A. taxiformis*, although it has been tested in *A. armata*, where it failed to induce tetrasporangium formation (Oza 1989; Mihaila et al. 2023). The observed success of *A. taxiformis* under an equinox photoperiod may reflect its adaptation to lower latitudes and its seasonal reproductive timing.

Based on regular monitoring data from local observatories (Nagasaki Prefecture, Stationary seawater temperature monitoring), sea surface temperatures in Nagasaki Prefecture, where our algal samples were collected, typically range from 20 to 25 °C between May and July and again between September and November (https://www.pref.nagasaki.jp/bunrui/shigoto-sangyo/suisangho/suisan-shiken-suishi-teichi-water-temperature/, accessed 19 April 2025). Considering the corresponding seasonal day length (10–12 h from September to November) and the 12.3-day induction period for a 50% probability of spore release at 25 °C (Fig. 2), sporulation in the field was most likely to occur between September and November. This autumnal sporulation pattern may be characteristic of Japanese *A. taxiformis* and is consistent with a previous report on tetraspore release observed in Mie Prefecture during that season (Chihara 1960).

As shown in Fig. 2, tetrasporophytes released spores earlier at 25 °C (12.3 ± 1.03 days) than that under the short photoperiod (15.1 ± 1.26 days), and the number of released tetraspores was also greater at 25 °C (6.69 ± 5.12 in the second week and 62.0 ± 48.9 in the third week) than under the short photoperiod (0.128 ± 0.198 in the second week and 3.66 ± 2.65 in the third week). These results suggest that temperature has a stronger influence on sporulation than photoperiod under controlled conditions. Therefore, we recommend that future protocols prioritize temperature regulation.

### The Growth and Differentiation of Gametophytes in Laboratory Conditions

Culturing gametophytes in artificial seawater (A/SW) resulted in growth retardation after 12 days (Fig. 3), suggesting that either excessive or deficient chemical components may have affected their development. Although the commercial artificial seawater product used in this study (Marine Art SF-1) has been successfully applied to other seaweeds such as *Ulva prolifera* and *Gayralia oxysperma* (Ichihara et al. 2019; Kinoshita et al. 2022), *A. taxiformis* showed limited growth in A/SW, indicating the possible absence of growth-promoting elements in the medium. Similar growth retardation was reported in *Lithophyllum okamurae* using the same A/SW commercial product (Yoshioka et al. 2020). Considering that nitrate and phosphate were enriched in A/SW and natural seawater (N/SW), nutrients other than nitrogen and phosphorus were absent in the A/SW.

Although the composition of the A/SW used in this study is known (Table S2), the chemical profile of the N/SW was not analyzed. Natural seawater typically contains various inorganic and organic compounds, including trace elements, which can vary depending on the season, location, depth, and treatment (Nozaki 1997). Therefore, it is likely that the N/SW used in this study provided sufficient nutrients for *A. taxiformis* growth. This is supported by the growth performance in mixed seawater (N + A/SW), which was lower than that in N/SW alone, likely because of dilution by A/SW (Fig. 3).

Interestingly, our preliminary comparison of the growth between gametophytes and tetrasporophytes in A/SW showed that gametophytes exhibited growth retardation after two weeks, whereas tetrasporophytes grew well in both N/SW and A/SW for four weeks (Fig. S6a). However, when tetrasporophytes were maintained in A/SW beyond the experimental period, they lost pigmentation and experienced biomass degradation after approximately three months (Fig. S6b). These results suggest that tetrasporophytes, like gametophytes, are also affected by the chemical composition of A/SW over the long term.

Notably, gametophytes showed comparable growth rates in both N/SW and A/SW during the first nine days (Fig. S4), indicating that early growth may rely on internally stored nutrients. Taken together, these findings suggest that both life stages of *A. taxiformis* are capable of growth in the short term under nutrient-limited conditions, but require certain elements present in N/SW for sustained growth and development.

Aeration promoted a significant increase in the fresh weight of the gametophytes (Fig. 4a). In addition to maintaining adequate levels of dissolved oxygen and carbon dioxide, aeration enhances water movement, which in turn reduces the thickness of the diffusion boundary layer and facilitates the uptake of nutrients, including carbon, nitrogen, and phosphorus, which are key elements for algal productivity (Hurd 2000; Msuya and Neori 2008). Recent studies have reported that water movement enhances the growth of both gametophytes and tetrasporophytes in *A. armata* (Mihaila et al. 2024; Hall et al. 2025). In addition to increased biomass, aerated gametophytes exhibited reduced moisture content (Fig. 4c), implying increased dehydrated biomass, such as starch, protein, and lipids. Bromoform, a hydrophobic halogenated compound, also accumulated at high concentrations under aerated conditions (Fig. 4d). Similar effects of aeration on biomass composition have been reported in *Ulva* sp., where higher aeration rates increase starch content (Traugott et al. 2020), and in the microalga *Scenedesmus dimorphus*, where reduced aeration limits lipid accumulation (Eustance et al. 2015). The observed increase in *A. taxiformis* gametophyte biomass under aeration was consistent with these findings.

*Asparagopsis* gametophytes exhibit a distinct shoot–rhizome structure, with erect main axes and pinnate lateral branches forming feathery fronds and densely branched rhizoidal stolons (Bonin and Hawkes 1987; HasLin and Pellegrini 2001). Aeration facilitated the development of these characteristic features, including fronds and entangled rhizomes. In contrast, the static culture led to the elongation of thin, sparsely branched axes, making it difficult to distinguish between the shoot and rhizome structures (Fig. 4b). Morphological traits (e.g., frond structure and rhizoidal stolons) were clearly observed in gametophytes grown in a 50-L aquarium with gentle aeration. In the growth experiment, gametophytes were cultured in 500-mL flasks that enabled the efficient circulation of the medium, but gentle aeration provided a low intensity of water motion in the stock culture of gametophytes in the aquarium. There was a noticeable difference in the water motion between the experimental flask and the stock aquarium.

In addition to water motion, algal density may play a role. In the growth experiment, the gametophyte biomass reached 2 g/L (fresh weight) after 28 days. Stocking density influences algal growth performance owing to self-thinning, which is induced by self-shading and nutrient limitation (Xiao et al. 2019). Thus, both water motion and biomass density likely influenced the growth and morphological differentiation of gametophytes in different culture vessels.

### The Bromoform Distribution and Emissions

The bromoform content in *A. taxiformis* under laboratory conditions has been studied on tetrasporophytes (Hargrave et al. 2024; Resetarits et al. 2024; Torres et al. 2024). In the present study, bromoform accumulation was observed in laboratory-cultured gametophytes (Figs. 4, 5, 6). Concentrations varied depending on culture conditions (15.3–33.5 mg·g⁻^1^ DW; Fig. 4d) and tissue type (5.27–17.8 mg·g⁻^1^ DW; Fig. 5c). These values are relatively high compared to those reported for wild-harvested gametophytes from other regions: 6.6–43.5 mg·g⁻^1^ DW (Paul et al. 2006a), 4.39 ± 0.07 mg·g⁻^1^ DW (Vucko et al. 2017), 19.2 ± 2.1 mg·g⁻^1^ DW (Magnusson et al. 2020), and 6.5 ± 1.0 and 8.5 ± 1.0 mg·g⁻^1^ DW in male and female gametophytes, respectively (Patwary et al. 2024). However, direct comparisons must be approached with caution, as bromoform levels vary depending on the analytical methods, geographic origin, culture conditions, and storage management (Vucko et al. 2017; Zanolla et al. 2022b; Hutchings et al. 2024).

In the previous studies on laboratory-cultured tetrasporophytes, higher bromoform concentrations have often associated with enhanced algal growth (Hargrave et al. 2024; Resetarits et al. 2024; Torres et al. 2024). Similarly, in our study, gametophytes grown under aeration exhibited both higher growth rates and increased bromoform content (Fig. 4), which is consistent with those findings in tetrasporophytes.

Even under identical extraction and GC–MS protocols, we observed apparent differences in the bromoform content between gametophytes from the aeration experiments (Fig. 4) and those from the stock aquarium used for tissue dissection (Fig. 5). In addition, the secretion experiment using the same stock-cultured gametophytes showed a mean bromoform content of 13.0 ± 3.56 mg·g⁻^1^ DW (Fig. 6a), consistent with the dissection experiment. These differences likely reflect environmental factors, such as water motion and algal density, both of which differed substantially between the 500-mL flask cultures and 50-L stock aquariums. Factors such as tidal currents and wave exposure may influence bromoform production in natural marine environments.

A previous study found that the bromoform was more concentrated in the branches than in the main axes or cystocarps of wild *A. armata* gametophytes (Vergés et al. 2008). Our study also found significantly higher concentrations in the lateral branches than in the main axes, even under laboratory conditions (Fig. 5c). This was probably due to the greater abundance of gland cells in the branch tissue (Fig. S7). In our analysis, rhizomes were defined as the basal portion of the thallus that remained after the shoot structures were removed (Fig. 5a). As such, gland cell-abundant branches may have been retained in the rhizome, potentially explaining the comparable bromoform concentrations between rhizomes and branches. Thus, the stolon axes likely possessed the same composition as the main frond axes. Indeed, rhizomes exhibited higher moisture content and lower bromoform content on a fresh weight basis than shoot lateral branches (Figs. 5b and S5), suggesting that rhizoidal stolons, except for branches, have properties that are more similar to the main axes than to lateral branches.

In the culture medium of tetrasporophytes, the bromoform concentration increased steadily over time (Fig. 6d), whereas no such trend was observed in gametophyte cultures (Fig. 6c). This suggested that tetrasporophytes continuously release bromoform into the medium, whereas gametophytes may only release it sporadically, possibly during physical stress, such as transfer to new media, or they degrade bromoform internally. The internal bromoform concentration in gametophytes also decreased over time (Fig. 6a). To date, no metabolic pathways for bromoform degradation have been described in seaweeds. However, Okuda et al. (2024) reported marine α- and γ-proteobacteria capable of degrading bromoform. Our culture experiments were not performed under axenic conditions. The presence of bromoform-degrading bacteria could have contributed to the reduction in the extracellular bromoform concentration.

The calculated bromoform release rates for gametophytes (28.128 ± 26.815 (± 8.085 SE) µg g^−1^ DW h^−1^) and tetrasporophytes (43.920 ± 20.743 (± 6.254 SE) µg g^−1^ DW h^−1^) were substantially higher than those previously reported for laboratory-cultured tetrasporophytes of *A. armata* (1.110 (± 0.393 SE) µg g^−1^ DW h^−1^; Paul et al. 2006a). These discrepancies likely reflect to not only species (i.e., *A. taxiformis* vs *A. armata*), but also differences in the incubation conditions (e.g. light intensity, photoperiod) and analytical protocols. Carpenter et al. (2000) reported a bromoform release of 45,200 pmol g^−1^ DW h^−1^ in *A. armata* collected in Ireland, which is equivalent to 11.423 µg g^−1^ DW h^−1^ and similar to our estimates.

Bromoform is a known inhibitor of microbial methanogenesis and has attracted attention as a bioactive component of livestock feed derived from *Asparagopsis* spp. (Glasson et al. 2022). It is a common halogenated metabolite in red algae (Laturnus 2001) and is particularly abundant in *Asparagopsis* because of its high accumulation in gland cells (Paul et al. 2006b; Romanazzi et al. 2021). While its ecological role in *A. taxiformis* is presumed to be defensive (e.g., against herbivory and biofouling), some studies have reported weak antibacterial activity compared to other halogenated compounds (e.g., dibromoacetic acid) and even an attractant effect on sea slugs (Hay and Fenical 1988; Paul et al. 2006a; Vergés et al. 2008). These findings imply that bromoform excretion may not function solely as a chemical defense mechanism in *Asparagopsis* spp.

Vanadium-dependent haloperoxidases (VHPOs) are the primary enzymes that catalyze halogenation reactions in marine algae (Wever and Horst 2013). The genome of *A. taxiformis* contains the marine bromoform biosynthesis locus (*mbb* locus), which includes three genes encoding VHPOs (*mbb1*, *mbb3*, and *mbb4*) and one gene encoding NAD(P)H oxidase (*mbb2*) (Thapa et al. 2020; Zhao et al. 2022). Thapa et al. 2020 demonstrated that recombinant Mbb1 and Mbb4 exhibited brominating activity for substrates, including pentane-2,4-dione, heptane-2,4,6-trione, and malonyl-S-acyl carrier proteins, producing the bromoform. Furthermore, genome annotation predicted the secretion of signal peptides in all three VHPOs, and proteomic analyses confirmed their presence in water-insoluble supernatants extracted from gametophytes and tetrasporophytes (Zhao et al. 2022; Patwary et al. 2023). Together with our findings, the leakage of bromoform likely resulted from secreted VHPO activity. However, the mechanisms regulating bromoform biosynthesis and emission in gametophytes and tetrasporophytes remain to be elucidated. This study highlights the different secretion patterns of bromoform between gametophytes and tetrasporophytes (Fig. 6), as well as apparent effects of culture conditions on intracellular bromoform accumulation (Fig. 5). These insights provide a valuable foundation for optimizing cultivation protocols and improving bromoform yields of *A. taxiformis* gametophytes.

## Conclusion

This study demonstrated that either an elevated temperature (25 °C) or a short photoperiod (8:16 L:D) successfully induced tetraspore release in *Asparagopsis taxiformis* collected from Japan. The released spores germinate and develop into juvenile gametophytes when cultured in natural seawater. In contrast, artificial seawater failed to support gametophyte growth beyond the first week, suggesting that some essential growth-promoting elements may be absent from the listed composition. Aeration facilitated the development of normal gametophyte morphology and enhanced bromoform accumulation, particularly in lateral branches. Furthermore, distinct bromoform secretion patterns were observed between gametophytes and tetrasporophytes. These findings contribute to developing a practical laboratory protocol for gametophyte seedling production and offer physiological insights essential for advancing aquaculture techniques for *A. taxiformis*.

## Supplementary Information

Below is the link to the electronic supplementary material.

Supplementary file1 (PDF 1258 KB)

## Data Availability

Data supporting the study’s findings is provided within the manuscript or supplementary information files. Detailed data can be accessed from the corresponding author upon reasonable request.
