# Supplementary material for: A Demonstration of Bromoform-Producing Gametophyte Culture for a Red Alga, Asparagopsis taxiformis in Laboratory Conditions
Source: Mar Biotechnol (NY). 2025 Jul 21;27(4):115. doi: 10.1007/s10126-025-10493-2 (PMC12277227; doi:10.1007/s10126-025-10493-2)
Supplement: Supplementary file 1 — Supplementary file1 (PDF 1258 KB) [file 10126_2025_10493_MOESM1_ESM.pdf]

Supplementary information: A demonstration of gametophyte culture for a red alga, *Asparagopsis taxiformis* in laboratory conditions

Marine biotechnology, 2025

Ryuya Matsuda*, Fishery division, Japan International Research Center for Agricultural Science (JIRCAS), 1-1 Owashi, Tsukuba, Ibaraki 305-8686, Japan

Kazuyoshi Kuwano, Faculty of Fisheries, Nagasaki University, 1-14 Bunkyo-Machi, Nagasaki 852-8521, Japan

*Corresponding author

Email: matsudar0337@jircas.go.jp

Phone: (+81) 29 838 6357

List of figures

Figure S1 Photographs of tetrasporophytes and gametophytes cultured under laboratory conditions.

(a) Stock cultures of tetrasporophytes. (b) Gametophytes grown in stock culture. (c) Male gametophytes with spermatangia. (d) Female gametophytes with cystocarps. (e) Mature tetrasporophytes with tetrasporangia (arrows). (f) Enlarged view of tetrasporangia. (g) Released tetraspores. (h) Germinated tetraspores.

Figure S2 Growth of tetrasporophytes during sporulation experiment.

Regression lines and 95% confidence intervals (grey region) were generated using linear model.

Figure S3 Effect of photoperiod on sporulation at 25 °C.

Tetrasporophytes were incubated under equinox daylength (12:12 L:D) and short daylength (8:16 L:D) at 25 °C. The mean ± SD of the first day of tetraspore release in each culture unit was calculated (n = 12).

Figure S4 Daily growth rates (DGR) of gametophytes cultured in different seawater media.

Boxplots show DGR values on days 6, 9, 12 and 33.

Figure S5 Bromoform content on a fresh weight basis in gametophytes.

Bromoform concentrations in each tissue type were calculated as in Fig. 5d of the main text. Statistical analysis was performed using GLM, followed by Tukey's HSD test for multiple comparisons (ns, not significant, $^{*}P \leq 0.05$, $^{***}P \leq 0.001$).

Figure S6 Comparison of growth between gametophytes and tetrasporophytes in

artificial seawater.

(a) Weekly changes in algal weight of gametophytes and tetrasporophytes monitored during a preliminary experiment (n = 3). The culture medium was refreshed every week. (b) Photograph of tetrasporophytes cultured in natural seawater (N/SW; left) and artificial seawater (A/SW; right) for over 3 months.

Figure S7 Representative microscopic images of lateral branch and main axis.

(a–c) Lateral branch. (d–f) Main axis. Images (c) and (f) are enlarged views of the framed area in (b) and (e), respectively. Arrowheads indicate gland cells.

List of tables

Table S1 Summary of tetraspore release experiments

Table S2 Chemical composition of commercial artificial seawater used in this study (Marine Art SF-1, Osaka Yakken Co., Ltd., Japan)

Figure S1

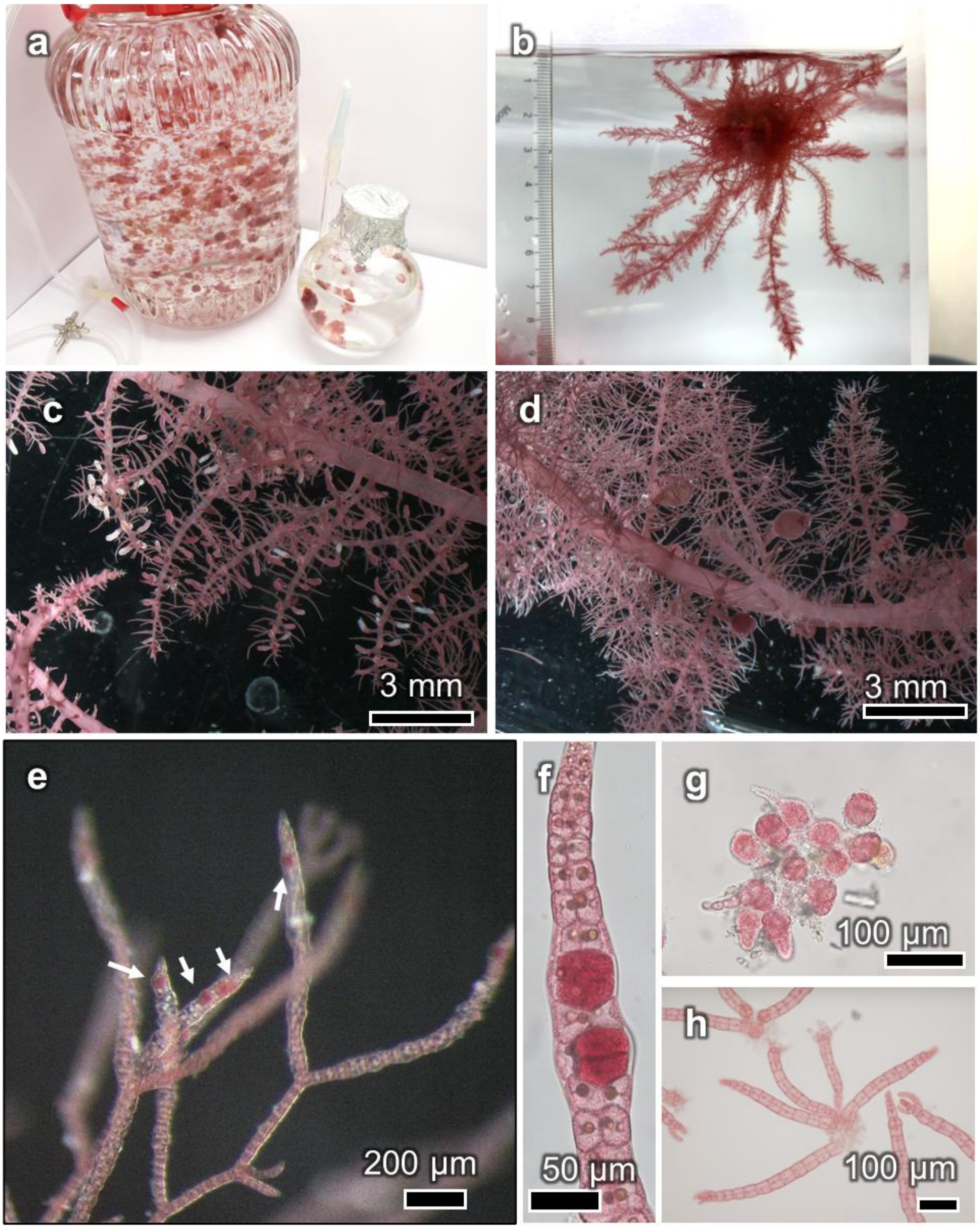

Figure S2

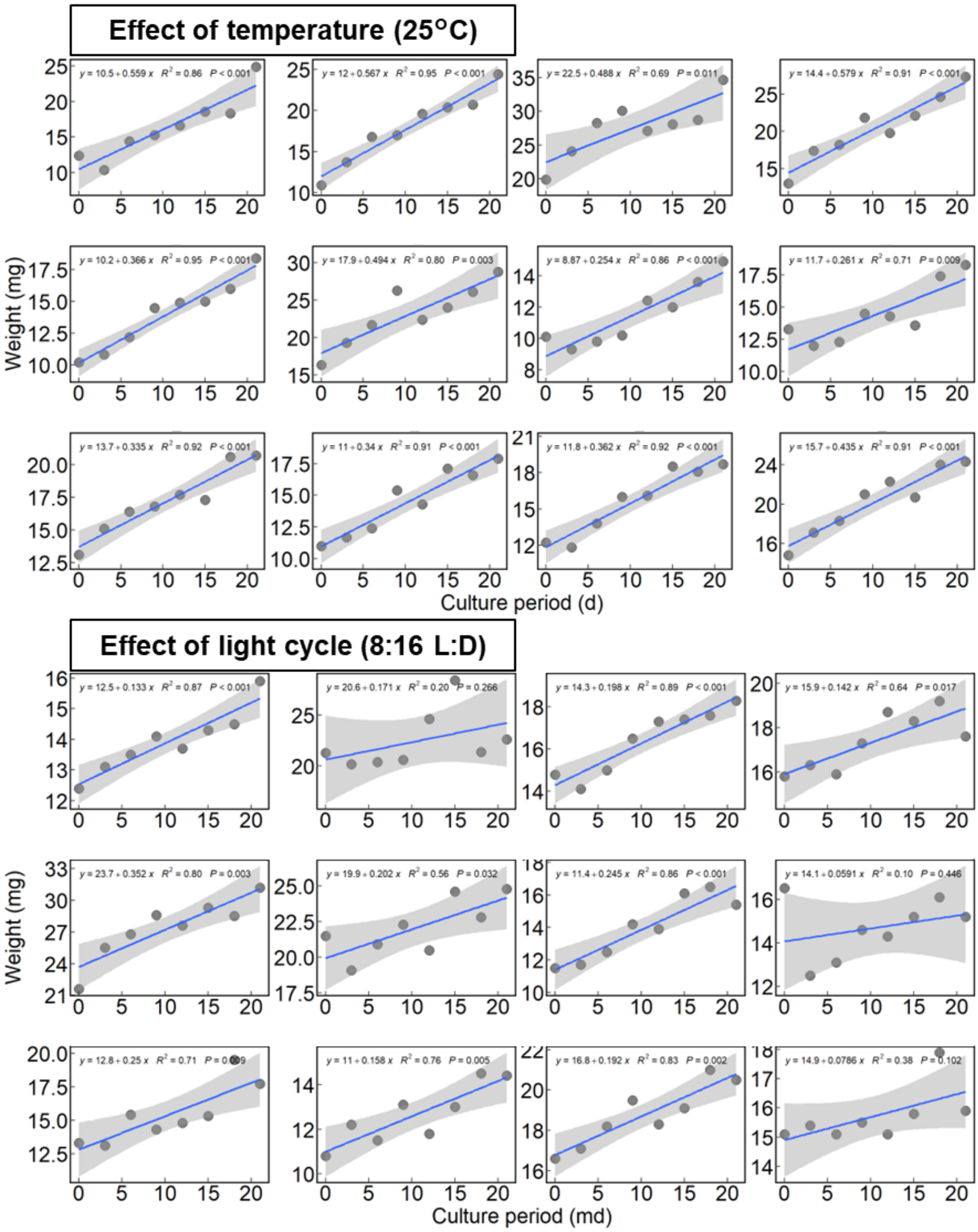
Effect of temperature (25°C)
y = 10.5 + 0.559 x R² = 0.86 P < 0.001
y = 12 + 0.567 x R² = 0.95 P < 0.001
y = 22.5 + 0.488 x R² = 0.69 P = 0.011
y = 14.4 + 0.579 x R² = 0.91 P < 0.001
y = 10.2 + 0.366 x R² = 0.95 P < 0.001
y = 17.9 + 0.494 x R² = 0.80 P = 0.003
y = 8.87 + 0.254 x R² = 0.86 P < 0.001
y = 11.7 + 0.261 x R² = 0.71 P = 0.009
y = 13.7 + 0.335 x R² = 0.92 P < 0.001
y = 11 + 0.34 x R² = 0.91 P < 0.001
y = 11.8 + 0.362 x R² = 0.92 P < 0.001
y = 15.7 + 0.435 x R² = 0.91 P < 0.001
Weight (mg)
Culture period (d)
Effect of light cycle (8:16 L:D)
y = 12.5 + 0.133 x R² = 0.87 P < 0.001
y = 20.6 + 0.171 x R² = 0.20 P = 0.266
y = 14.3 + 0.198 x R² = 0.89 P < 0.001
y = 15.9 + 0.142 x R² = 0.64 P = 0.017
y = 23.7 + 0.352 x R² = 0.80 P = 0.003
y = 19.9 + 0.202 x R² = 0.56 P = 0.032
y = 11.4 + 0.245 x R² = 0.86 P < 0.001
y = 14.1 + 0.0591 x R² = 0.10 P = 0.446
y = 11 + 0.158 x R² = 0.76 P = 0.005
y = 16.8 + 0.192 x R² = 0.83 P = 0.002
y = 14.9 + 0.0786 x R² = 0.38 P = 0.102
Weight (mg)
Culture period (md)

Figure S3

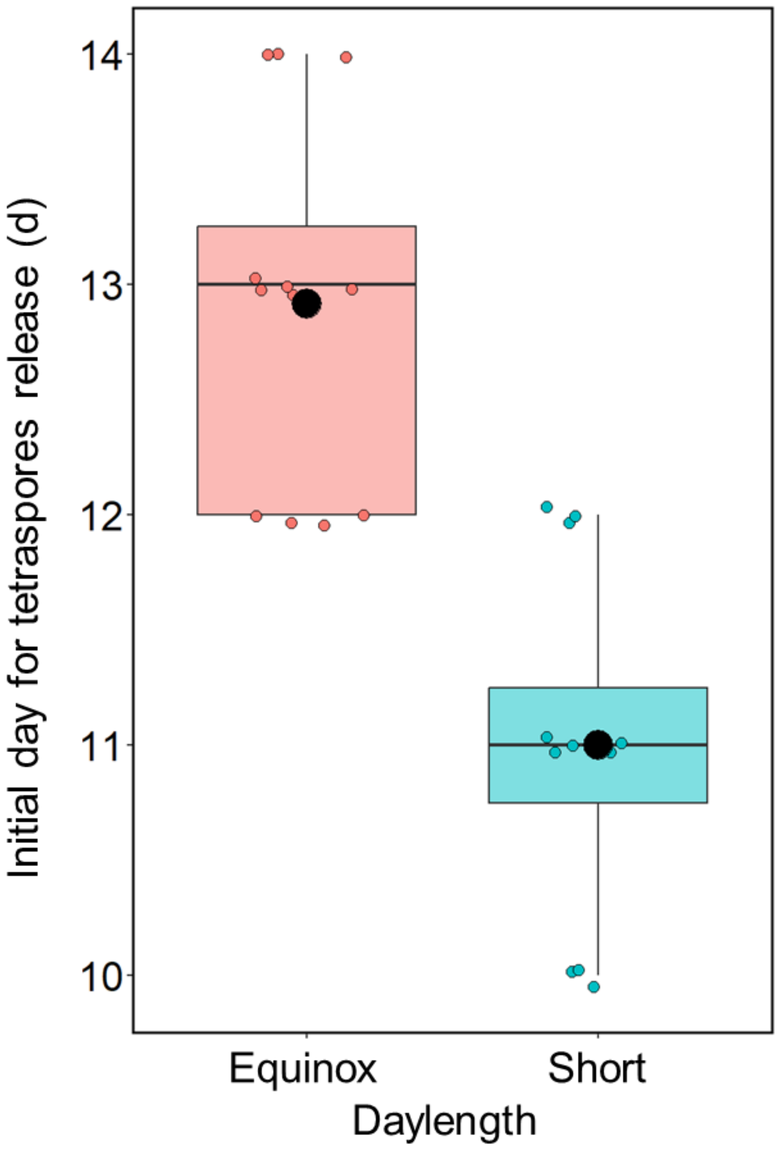

14
13
12
11
10
Initial day for tetraspores release (d)
Equinox
Short
Daylength

Figure S4

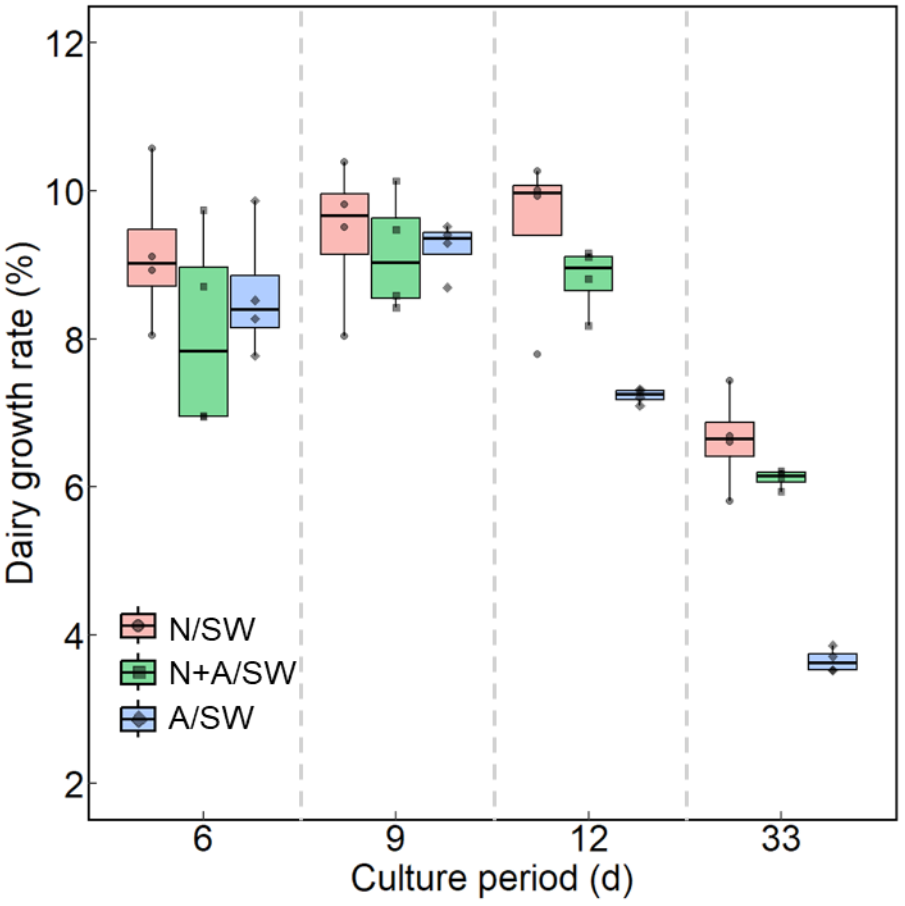

12
10
8
6
4
2
Dairy growth rate (%)
N/SW
N+A/SW
A/SW
6
9
12
33
Culture period (d)

Figure S5

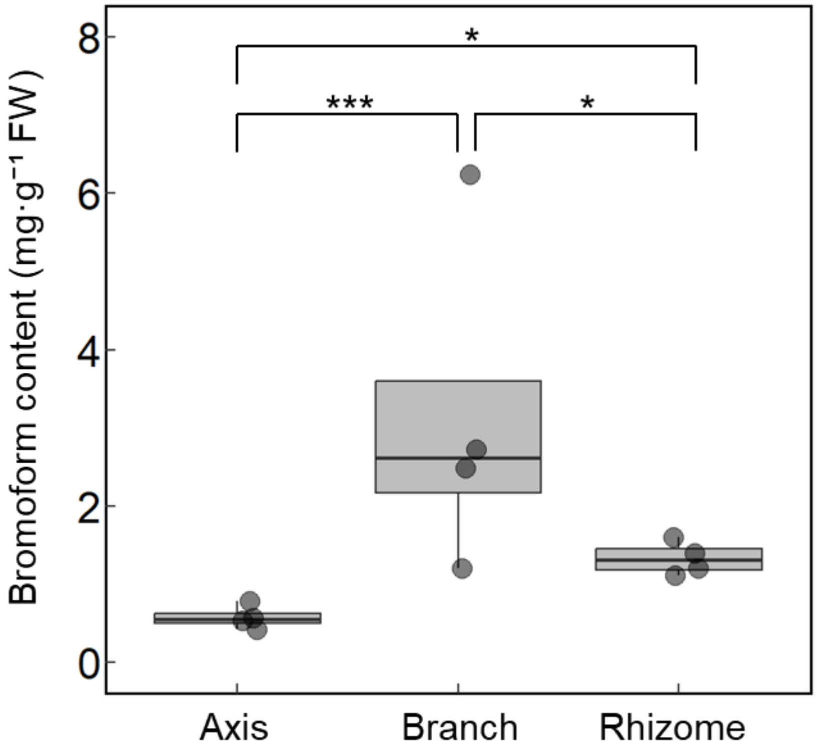

Figure S6

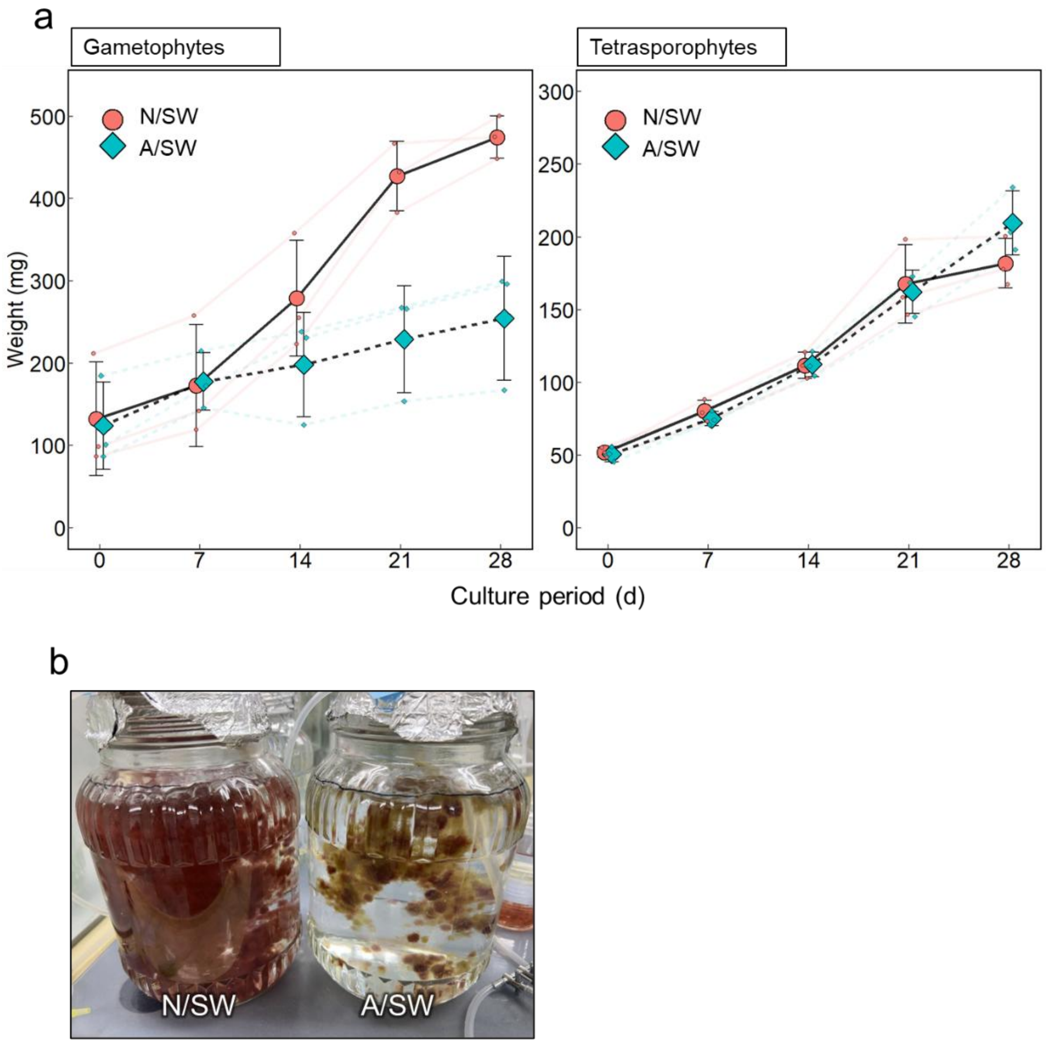
a
Gametophytes
Tetrasporophytes
N/SW
A/SW
Weight (mg)
Culture period (d)
0
100
200
300
400
500
50
150
250
7
14
21
28
b
N/SW
A/SW

Figure S7

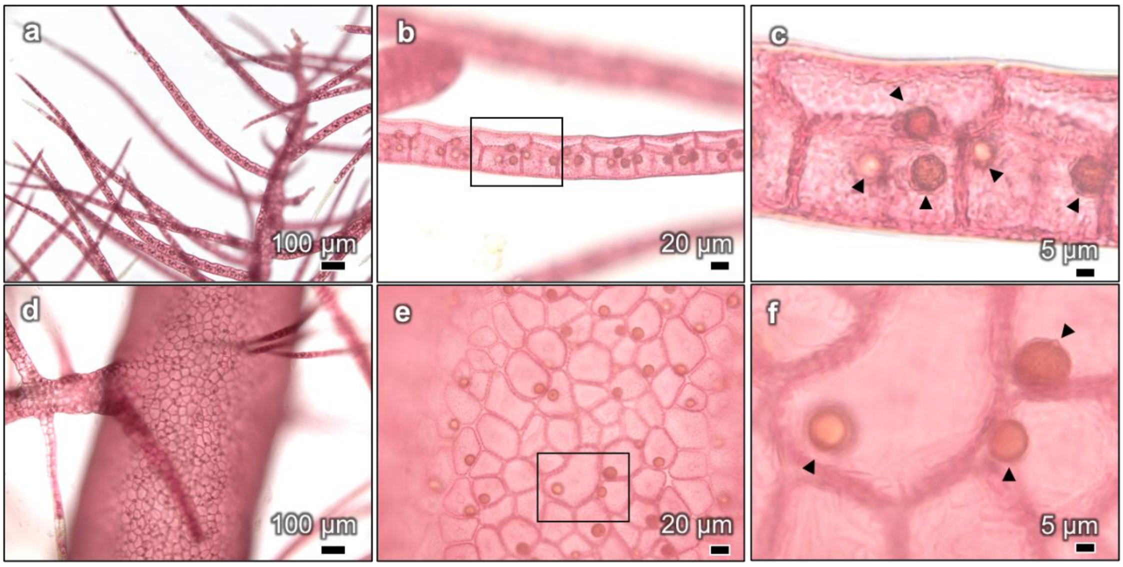

Table S1

Species	Collection site	Conditions for stock culture		Conditions for induction experiment		Period until release of tetraspore	Reference
		Temperature (°C)	Daytime (h)	Temperature (°C)	Daytime (h)		
A. armata	Roscoff, France	15	10	15	6 – 8	ND	Oza 1989
A. armata	Messina, Sicilym Italy	16	16	17	8 – 9	Within 5 weeks	Guiry and Dawes 1992
				17 – 21	8	Within 5 weeks	
	New Quay, Co Ckare, Ireland	16	16	17	8 – 9	Within 5 weeks	
				17 – 21	8	Within 5 weeks	
	Sorrento, Victoria, Australia	17	14	17	8	Within 5 weeks	
				13 – 17	8	Within 5 weeks	
A. armata	Sorrento, Victoria, Australia	15	16	11 – 21	8	Within 5 weeks	Ní Chualáin et al. 2004
	Isla Robinson Crusoe, Chile			17 – 19	8	Within 5 weeks	
	New Quay, Co Ckare, Ireland	20	16	17 – 19	8	Within 5 weeks	
	Messina, Sicilym Italy			17 – 21	8	Within 5 weeks	
	Villefranche-sur-mer, France			17 – 21	8	Within 5 weeks	
	Puerto di Porticella, Ovin˜ana, Asturias, Spain			17 – 23	8	Within 5 weeks	
	Artedo, Spain			15 – 21	8	Within 5 weeks	
	Horse Ledges, Shanklin, Isle of Wight, UK			15 – 21	8	Within 5 weeks	
	Portelet Bay, Jersey, Channel Is, UK			17 – 19	8	Within 5 weeks	
	L'Archirondel, Jersey, Channel Is, UK			11 – 21	8	Within 5 weeks	
A. taxiformis	Key Largo, Florida, USA	25	16	25 – 29	8	Within 5 weeks	
	La Paugain, Puerto Rico			23 – 25	8	Within 5 weeks	
	Hawaii, USA			17 – 29	8	Within 5 weeks	
	Puerto Morales, Quintana Roo, Yucatan, Mexico			23 – 25	8	Within 5 weeks	
	Villa St. Giovanni, Messina, Sicily, Italy			21 – 27	8	Within 5 weeks	
	Komesu, Itoman, Okinawa I., Okinawa Pref., Japan			21 – 27	8	Within 5 weeks	
	Hinomi-saki, Taisha, Shimane Pref., Japan			17 – 27	8	Within 5 weeks	
	Komesu, Itoman, Okinawa I., Okinawa Pref., Japan			21 – 29	8	Within 5 weeks	
	Hon Tre, Nha Thrang, Khan Hoa Province, Vietnam			23	8	Within 5 weeks	
A. armata	Matheson Bay, Leigh, New Zealand	18	12	15 – 18	8	Within 3 weeks	Mihaila et al. 2023
A. taxiformis	Gladstone Harbour, Queensland, Australia	19	16	19 – 25	8	Within 1–2 week(s)	Theobald et al. 2024
A. taxiformis	Minamishimabara, Nagasaki, Japan	20	12	25	12	12.3 ± 1.03 days	This study
A. taxiformis	Minamishimabara, Nagasaki, Japan	20	12	20	8	15.1 ± 1.26 days	This study

Table S2

Chemical compound	Chemical formula	MW	Cont per L	Concentration
Sodium Chloride	$NaCl$	58.44	22.1 g	378.2 mM
Magnesium chloride	$MgCl_2 \cdot 6H_2O$	203.3	9.9 g	48.7 mM
Calcium chloride	$CaCl_2 \cdot 2H_2O$	147.01	1.5 g	10.2 mM
Sodium sulfate	Na_2SO_4	142.04	3.9 g	27.5 mM
Potassium chloride	KCl	74.5513	0.61 g	8.2 mM
Sodium bicarbonate	$NaHCO_3$	84.007	0.19 g	2.3 mM
Potassium bromide	KBr	119.002	96 mg	806.7 µM
Borax	$Na_2B_4O_7 \cdot 10H_2O$	381.37	78 mg	204.5 µM
Strontium chloride	$SrCl_2$	158.53	13 mg	82.0 µM
Sodium fluoride	NaF	41.98817	3 mg	71.4 µM
Lithium chloride	$LiCl$	42.394	1 mg	23.6 µM
Potassium iodide	KI	166.0028	81 µg	487.9 nM
Manganese chloride	$MnCl_2 \cdot 4H2O$	197.91	0.6 µg	3.0 nM
Cobalt chloride	$CoCl_2 \cdot 6H_2O$	129.839	2 µg	15.4 nM
Aluminum chloride	$AlCl_3 \cdot 6H_2O$	241.43	8 µg	33.1 nM
Ferric chloride	$FeCl_3 \cdot 6H_2O$	270.29	5 µg	18.5 nM
Sodium tungstate	$Na_2WO_4 \cdot 2H_2O$	329.85	2 µg	6.1 nM
Ammonium molybdate	$(NH_4)_6Mo_7O_{24} \cdot 4H_2O$	1235.92	18 µg	14.6 nM
